# Prevalence, Molecular Typing, and Determination of the Biofilm-Forming Ability of *Listeria monocytogenes* Serotypes from Poultry Meat and Poultry Preparations in Spain

**DOI:** 10.3390/microorganisms7110529

**Published:** 2019-11-05

**Authors:** Carlos Alonso-Calleja, Sara Gómez-Fernández, Javier Carballo, Rosa Capita

**Affiliations:** 1 Department of Food Hygiene and Technology, Veterinary Faculty, University of León, E-24071 León, Spain; carlos.alonso.calleja@unileon.es (C.A.-C.); sgomef01@gmail.com (S.G.-F.); 2 Institute of Food Science and Technology, University of León, E-24071 León, Spain; 3 Area of Food Technology, University of Vigo, E-32004 Ourense, Spain; carbatec@uvigo.es

**Keywords:** *Listeria monocytogenes*, serotypes, ribotypes, lineages, biofilms, poultry

## Abstract

A study was undertaken of the presence of *Listeria monocytogenes* in 260 samples of poultry meat obtained from retail outlets in northwestern Spain. *L. monocytogenes* was detected in 20 samples (7.7%). Twenty strains (one strain per positive sample) were characterized. The strains belonged to 10 serotypes: 1/2a (2 strains), 1/2b (2), 1/2c (2), 3a (1), 3b (2), 3c (2), 4a (2), 4b (4), 4c (1), and 4d (2). Cluster analysis (ribotyping; *Eco*RI) showed a strong genetic relationship between strains isolated from samples coming from different outlets. Ribotyping permitted some isolates of the same serotype to be differentiated, which points to the possible usefulness of this technique in the epidemiological surveillance of *L. monocytogenes*. All strains formed biofilm on polystyrene, as shown by confocal laser scanning microscopy. The biovolume (between 621.7 ± 36.0 µm^3^ and 62,984.0 ± 14,888.2 µm^3^ in the observational field of 14,161 μm^2^), percentage of surface coverage (from 2.17 ± 0.84% to 94.43 ± 3.97%), roughness (between 0.399 ± 0.052 and 0.830 ± 0.022), and maximum thickness (between 9.00 ± 0.00 µm and 24.00 ± 14.93 µm) of biofilms varied between strains (*p* < 0.05). These results expand knowledge of the characteristics of *L. monocytogenes* isolates from poultry.

## 1. Introduction

*Listeria monocytogenes* is a very widespread Gram-positive bacterium [1]. It is responsible for human listeriosis, a food-borne infection that in certain groups in the population (pregnant women, children, the elderly, and individuals with immunodepression) is linked to miscarriages or fetal death, sepsis, pneumonia, and serious infections of the central nervous system [2]. The prevalence of listeriosis is low; a total of 2480 confirmed cases of invasive human listeriosis were reported in the European Union in 2017, equating to 0.48 cases per 100,000 individuals. However, it is a worrying infection, since it is the food-borne illness with the highest rate of lethality, at 13.8% in the E.U. in 2017 [3].

*L. monocytogenes* is able to survive and multiply in adverse environmental conditions, such as low pH values and high salt concentrations, which are frequent in food-processing environments. The fact that it is a facultative anaerobe and psychotropic makes it difficult to control within the food industry [4]. This microorganism tends to maintain itself in the context of food-processing, particularly in places that cleaning and disinfecting personnel find it hard to access, such as drains, the rollers of conveyor belts, or cracked rubber seals around doors [5]. At times, *L. monocytogenes* can be repeatedly isolated in the same facility over a period of months or years, although it can also appear as the consequence of sporadic contamination [6]. It is believed the principal reason for the persistence of *L. monocytogenes* in these contexts is its ability to form biofilms, as such structures increase the microorganisms’ resistance to cleaning and disinfection procedures [7]. Moreover, at advanced stages in the development of biofilm, the bacteria can colonize and contaminate other surfaces [8]. The presence of biofilms in the food industry increases the risk of contamination of foodstuffs. It is estimated that over 60% of outbreaks of food-borne illnesses are associated with the presence of biofilms [9].

Confocal laser scanning microscopy (CLSM) is currently one of the most widely used tools for studying the structure of biofilms, since it allows in situ investigation of such structures through the use of specific fluorescent probes. Furthermore, the highly developed software for image analysis offers the possibility of obtaining detailed quantitative structural parameters for biofilms. To this end, CLSM does not just provide qualitative information about the structure of biofilm, it makes it feasible to characterize it in detail with numerical data and allows statistical analyses to be performed [10]. 

Discrimination between strains of *L. monocytogenes* is necessary when epidemiological studies are being carried out, as well as being of interest for monitoring sources of microbial contamination in food-processing plants. Serotyping, based on the presence of different somatic and flagellar antigens, has habitually been used as a method for typing strains of *L. monocytogenes*. Thirteen serotypes have been identified for this bacterium, comprising 1/2a, 1/2b, 1/2c, 3a, 3b, 3c, 4a, 4ab, 4b, 4c, 4d, 4e, and 7 [1]. Thanks to variations in their ecology, genomic content, and recombination rates, serotypes of *L. monocytogenes* are grouped in four distinct evolutionary lineages (I to IV) [2].

The limited range of serotypes involved in processes of human listeriosis (serotypes 1/2a, 1/2b, and 4b are responsible for approximately 95% of cases of human illness worldwide) [11] and the generally low number of serotypes detected in foodstuffs cause serotyping to be of limited usefulness. On occasion, it is necessary to make use of other methods permitting better discrimination between strains. Various different molecular techniques have been employed for this purpose over recent decades. The technique of ribotyping has been used frequently in the last few years to type different pathogenic microorganisms, including *L. monocytogenes* [12].

The aims of the work reported here were: (1) to gain a knowledge of the prevalence, serotypes and biofilm-forming ability of *L. monocytogenes* isolates from samples of poultry obtained from retail outlets in northwestern Spain; (2) to determine the usefulness of the ribotyping technique in discriminating between strains of *L. monocytogenes* of the same serotype; and (3) to study the possible utility of structural parameters of biofilms (biovolume, percentage of surface covered, maximum thickness, and roughness) in typing and discriminating between the various strains of *L. monocytogenes*.

## 2. Materials and Methods

### 2.1. Sampling Procedure

A total of 260 samples (each weighing about 250 g) of raw poultry (chicken, turkey, or both), were collected from twenty retail outlets (10 to 15 samples were obtained from each outlet) in the Province of León in northwestern Spain. Three or four visits were made to each establishment (three to five samples were collected on each visit). Samples included drumsticks (50 samples), wings (50), minced meat (40), red sausages (20), white sausages (20), hamburgers (20), meatballs (20), nuggets (20), escalope (10), and crepes (10). Each sample was placed in a separate sterile plastic bag, transported in an ice chest to the laboratory immediately after collection, and tested upon arrival or stored at 3 °C for no longer than 4 h.

### 2.2. Microbiological Analysis

The UNE-EN ISO 11290-1 method was used for detecting *L. monocytogenes* [13]. Samples (each weighing 25 g) were homogenized for two minutes in a Masticator (IUL, Barcelona, Spain) with 225 mL of half-Fraser broth (Oxoid Ltd., Hampshire, England). For drumsticks and wings, 25 g portions of the skin (from one drumstick or several wings in the same batch) were cut off aseptically. For sausages, hamburgers, meatballs, nuggets, escalope, and crepes, the 25 g samples were taken from two or more pieces in the same batch. After incubation at 30 °C for 24 h, 0.1 mL of the diluted sample was inoculated into tubes with 10 mL of Fraser broth (Oxoid) for secondary enrichment. After further incubation at 37 °C for 24 h, cultures were streaked onto chromogenic *Listeria* agar and onto PALCAM agar (Oxoid), these being incubated at 37 °C for 24 to 48 h.

For each sample, three typical colonies were characterized initially on the basis of Gram stain, catalase and oxidase reactions, typical umbrella motility, tumbling motility, nitrate reduction, Voges–Proskauer reaction, H_2_S production, beta-hemolysis and fermentation of glucose, mannitol, rhamnose, and xylose [14]. Presumptive *L. monocytogenes* strains were inoculated into micro-tubes of API *Listeria* (bioMérieux, Marcy L’Etoile, France) in accordance with the manufacturer’s instructions. The results were compared with information from the computer-aided database APIWEB^TM^ software (bioMérieux). One strain of *L. monocytogenes* was selected from each positive sample to be subjected to the tests listed below.

### 2.3. Serotyping and Ribotyping

Strains were stored at −50 °C in tryptone soya broth (TSB; Oxoid) supplemented with 20% (*v/v*) of glycerol. The frozen cells were sub-cultured in TSB at 37 °C for 24 h. These cultures were then streaked onto tryptone soya agar (TSA; Oxoid) plates, incubated for 24 h at 37 °C and then kept at 4 °C.

Serotyping on the basis of agglutination in the presence of antibodies reacting specifically with somatic (O) and flagellar (H) antigens, was carried out using a Seiken *Listeria* antisera kit (Denka Seiken Co., Tokyo, Japan), in accordance with the manufacturer’s instructions. Twelve antisera were used, eight for determining the somatic (O) antigens I/II, I, IV, V/VI, VI, VII, VIII, and IX, and four for the flagellar (H) antigens A, AB, C, and D. Strains were grouped into lineages on the basis of their serotype: lineage I, comprising serotypes 1/2b, 3b, 3c, and 4b, lineage II with serotypes 1/2a, 1/2c, and 3a, and lineages III and IV comprising serotypes 4a, 4c, and some atypical strains of serotype 4b [2,15,16].

Isolated colonies from TSA (Oxoid) plates freshly sub-cultured for 18–20 h were used for ribotyping. The automated RiboPrinter^®^ microbial characterization system (DuPont Qualicon, Wilmington, DE, USA) was used in accordance with the manufacturer’s instructions to perform ribotyping. Lyses of target cells with release of cellular DNA, *Eco*RI digestion of the chromosomal DNA, separation of the resulting fragments by agarose gel electrophoresis, and hybridization with a chemo-luminescent-labeled DNA probe containing the *E. coli* ribosomal RNA operon were carried out in 8 h.

### 2.4. Study of Biofilms

The formation and structure of biofilms were studied by a method previously described, with slight modifications [7]. Strains were cultured in TSB at 37 °C for 24 h. Decimal dilutions in TSB were prepared from these cultures until a concentration of approximately 10^5^ cfu/mL was reached. A volume of 250 µL was added to the wells of a sterile polystyrene microtiter plate (Matrix 96-well Polystyrene Flat Bottom microplates; Thermo Fisher Scientific, Rochester, NY, USA). After an hour of incubation at 37 °C, sufficient time to permit adhesion of bacterial cells to the surface of the wells, these were rinsed with a NaCl solution 150 mM to eliminate any bacteria that had not adhered, and then were refilled with 250 µL of TSB. The plate was next incubated at 37 °C for 24 h to allow the development of biofilms. Once this period had elapsed, the wells were once again washed with 150 mM NaCl. For staining with fluorescent dye, a volume of 1.25 µL of SYTO 9 (Invitrogen, Barcelona, Spain) was added to 1000 µL of TSB, and 250 μL of this solution was put into each well. The plate was then incubated in the dark at 37 °C for 20 min to enable fluorescent labelling of the bacteria.

The following procedure was used to acquire images with the aid of a Nikon Eclipse TE 2000-U confocal scanning laser microscope using the EZ-C13.60 program (Nikon Instruments Inc., Melville, NY, USA). All the biofilms were scanned at 400 Hz, using a 40× objective lens with a 488-nm argon laser set at 90% intensity. Three stacks of horizontal plane images (512 × 512 pixels, corresponding to an area of 119 × 119 µm) with a z step of 1 µm were acquired for each biofilm, using different areas in the well. Three independent experiments were performed for each condition, on different days.

### 2.5. Data Analysis

The number, position, and relative intensities of rRNA operon-specific DNA fragments were estimated automatically by RiboPrinter analysis software, and a digital record of 256 numerical values for each sample was produced. Linkage distances between patterns were estimated by means of Pearson’s correlation coefficient, and isolates were clustered by means of Ward’s method, using the Statistica 8.0 software package (Statsoft Inc., Tulsa, OK, USA). Groupings were formed and isolates with 0.1 linkage distance (1-Pearson’s r) assigned to them. This threshold was established from a cluster analysis of ribotyping profiles for three *L. monocytogenes* collection strains in three different gels.

Three-dimensional images of the biofilms were reconstructed by means of the IMARIS 9.1 program (Bitplane AG, Zurich, Switzerland) and quantitative structural parameters (biovolume, percentage surface coverage, roughness, maximum thickness) were calculated. The biovolume represented the overall volume of cells (μm^3^) in the observation field (14,161 μm^2^) and provided an estimate of the biomass in the biofilm. Surface coverage (%) reflected the efficiency of substratum colonization by the populations of bacteria. Roughness provided a measure of how much the thickness of the biofilm varied and was thus an indicator of biofilm heterogeneity [17]. A roughness with a value of zero indicates a biofilm of uniform thickness, and a value close to 1 describes a patchy biofilm. The maximum thickness (μm) of biofilms was determined directly from the confocal stack images.

Tree diagrams were drawn up (Euclidean distance, unweighted pair-group with arithmetic mean; Statistica^®^ 8.0) to group strains on the basis of details of the structural parameters of biofilms. Quantitative structural parameters for biofilms were analyzed by analysis of variance (ANOVA) techniques, with means being separated by Tukey’s test utilizing the Statistica^®^ 8.0 software package (StatSoft Inc., Tulsa, OK, USA). Significant differences were established for a probability level of 5% (*p* < 0.05).

## 3. Results

### 3.1. Prevalence of L. monocytogenes in Poultry

*L. monocytogenes* was detected in 20 of the 260 samples analysed (7.7%). These positive samples came from seven establishments (35% of the outlets sampled). These were outlet 2 (strains 1, 4, and 7), outlet 8 (strains 2 and 12), outlet 9 (strains 3, 5, 6, and 8), outlet 11 (strains 9, 10, 11, and 20), outlet 15 (strains 15, 18, and 19), outlet 18 (strains 13 and 14), and outlet 19 (strains 16 and 17). *L. monocytogenes* was found on drumsticks (5 strains; 10% of drumstick samples), on wings (2; 4%), in minced meat (6; 15%), hamburgers (3; 15%), meatballs (1; 5%), nuggets (1; 5%), escalope (1; 10%) and crepes (1; 10%).

### 3.2. Serotyping and Ribotyping of L. monocytogenes

**Figure 1:**
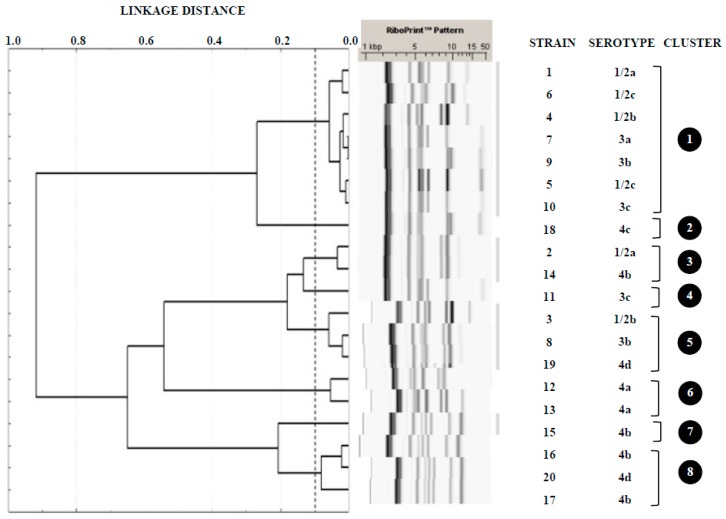
Tree diagram showing cluster analysis of RiboPrinter^®^ fingerprints from *Listeria monocytogenes* isolates from poultry. This was produced using Pearson’s correlation coefficient and Ward’s clustering method.

The twenty strains of *L. monocytogenes* studied were assigned to 10 different serotypes: 1/2a (2 strains; 10% of all isolates), 1/2b (2; 10%), 1/2c (2; 10%), 3a (1; 5%), 3b (2; 10%), 3c (2; 10%), 4a (2; 10%), 4b (4; 20%), 4c (1; 5%), and 4d (2; 10%). Figure 1 shows the clusters obtained with the restriction enzyme *Eco*RI from ribosomal DNA of *L. monocytogenes*, indicating the strains and serotypes comprised in them. Strains were grouped into eight different clusters (1 through to 8), three of which, numbers 2, 4, and 7, included just one single strain. The predominant cluster (1) brought together seven strains (from six different serotypes), whilst the remaining four (3, 5, 6, and 8) included either two or three strains each, mostly belonging to differing serotypes. In this work, ribotyping allowed discrimination of strains of serotypes 1/2a (clusters 1 and 3), 1/2b (clusters 1 and 5), 3b (clusters 1 and 5), 3c (clusters 1 and 4), 4b (clusters 3, 7 and 8), and 4d (clusters 5 and 8).

### 3.3. Architecture and Structural Parameters of Biofilms

**Figure 2:**
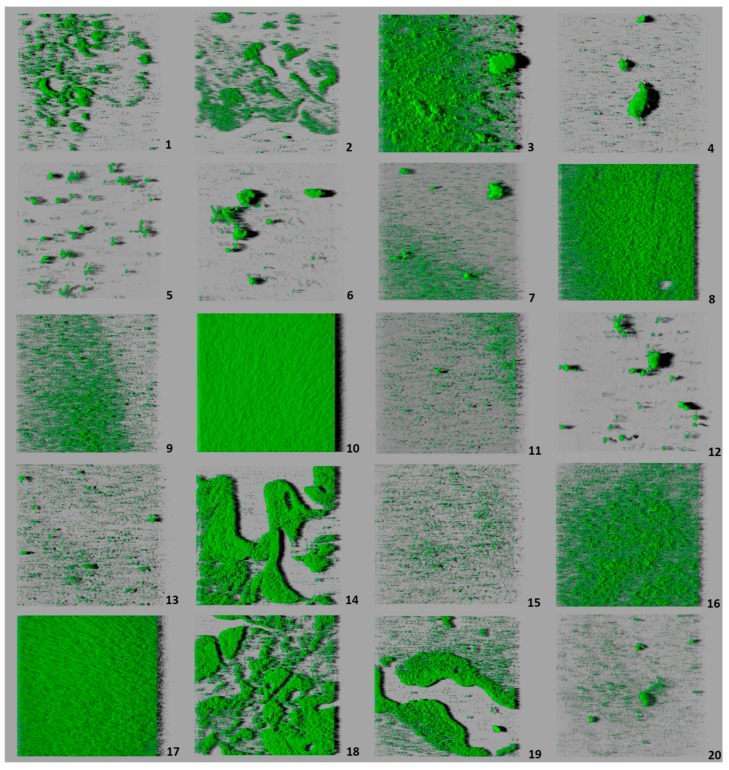
Three-dimensional projections of structures of biofilms obtained from one-micron optical sections on the *z*-axis acquired through confocal laser scanning microscopy. These images represent an overhead view of the biofilms formed by 20 strains of *Listeria monocytogenes*, with virtual projection of shadows to the right. Each square represented had length of side of 119 μm. Strains: 1 (serotype 1/2a), 2 (1/2a), 3 (1/2b), 4 (1/2b), 5 (1/2c), 6 (1/2c), 7 (3a), 8 (3b), 9 (3b), 10 (3c), 11 (3c), 12 (4a), 13 (4a), 14 (4b), 15 (4b), 16 (4b), 17 (4b), 18 (4c), 19 (4d), 20 (4d).

All the strains of *L. monocytogenes* were able to form biofilm on polystyrene, with considerable variations being observed from one strain to another (Figure 2). Table 1 gives the values for the structural parameters of biofilms from each of the strains tested. Table 2 shows the values obtained for the set of strains of each serotype. Strain 10 (serotype 3c) produced a biofilm with a larger biovolume (62,984.0 ± 14,888.2 µm^3^) than the remaining strains. These had from 621.7 ± 36.0 µm^3^ to 28,184.0 ± 21,540.5 µm^3^, with no significant differences found between them, despite the broad range of values obtained. When grouped by serotypes, strains of serotype 3c (lineage I) showed a trend to have the largest biovolume and those of serotype 1/2c (lineage II) the smallest (Table 2).

When strains were considered individually, a high level of correlation (r = 0.940, *p* < 0.01) was found between the parameters biovolume and percentage of surface covered. Strain 10 (serotype 3c) had the highest value for this parameter (94.43 ± 3.97%), indicating that biofilm covered the greater part of the surface observed (Figure 2). For the remainder of the strains, the percentage of surface coverage varied from 2.17 ± 0.84% (strain 6; serotype 1/2c) to 66.49 ± 30.47% (strain 17, serotype 4b).

When strains were looked at with grouping into serotypes, isolates of serotype 3c (lineage I) were those whose biofilm had the highest percentage of surface covered. They presented significant differences with regard to this parameter from the greater part of the serotypes.

There was a moderate negative correlation (r = −0.492, *p* < 0.05) between roughness and biovolume, and a strong negative correlation (r = −0.773, *p* < 0.01) between roughness and percentage of surface coverage. This means that the strains with the lowest values for biovolume and percentage of surface covered formed the most heterogeneous biofilms. Strain 10 (serotype 3c) confirmed this fact, because it presented the highest values for biovolume and percentage of surface covered, while simultaneously having the lowest value for roughness (0.399 ± 0.052) in the whole set of strains tested. Strain 12 (serotype 4a) had the highest value for roughness (0.830 ± 0.022). With regard to serotypes, 1/2c (lineage II) and 4a (lineage III/IV) were those which formed biofilms with the highest values for roughness, and were thus the most heterogeneous.

Maximum thickness showed no correlation with biovolume or percentage of surface covered (*p* > 0.05), but it did correlate positively with roughness (r = 0.849, *p* < 0.01). These results indicate that the thicker the biofilm attained, the greater the degree of heterogeneity it presented.

Strains 5 (serotype 1/2c) and 12 (serotype 4a) formed the biofilms with the greatest maximum thickness (24.00 ± 14.93 µm and 24.00 ± 5.00 µm, respectively). These two strains also presented high values for roughness, confirming the correlation between these two parameters. The biofilms of strain 15 (serotype 4b) had the lowest values for maximum thickness (9.00 ± 0.00 µm). In respect of serotypes, serotypes 1/2c (lineage II) and 4a (lineage III/IV) were those which formed biofilm with the greatest thickness, presenting significant differences with regard to serotypes from lineage II (1/2a) and lineage I (3b and 4b). It should be noted that because the low number of strains tested here, additional data are required to confirm the differences observed between serotypes.

### 3.4. Grouping of Strains by Structural Parameters of Biofilms

**Figure 3:**
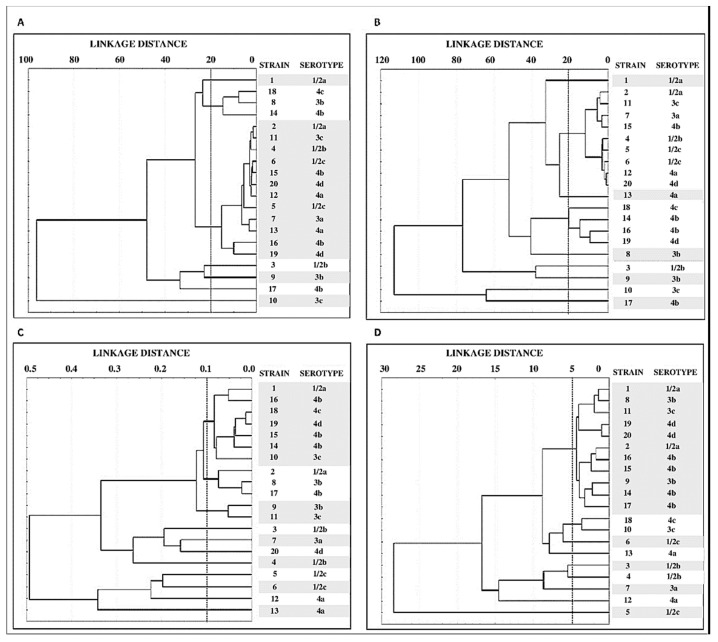
Tree diagrams showing grouping of values for quantitative parameters of biofilms. These are biovolume (**A**) percentage of surface covered (**B**) roughness (**C**) and maximum thickness (**D**) corresponding to 20 strains of *Listeria monocytogenes* taken from meat (Euclidean distance, unweighted pair-group average).

Figure 3 shows the tree diagrams obtained from the values of structural parameters (biovolume, percentage of surface coverage, roughness, and maximum thickness). On the basis of biovolume data (Figure 3A), strains fell into 7 clusters, one made up of 3 strains (8, 14, and 18) and another comprising 12 strains (2, 4, 5, 6, 7, 11, 12, 13, 15, 16, 19, and 20). The remainder of the strains (1, 3, 9, 10, and 17) were not able to be grouped.

Use of the data for percentage of surface covered (Figure 3B), the strains grouped into nine clusters, one of them composed of nine strains (2, 4, 5, 6, 7, 11, 12, 15, and 20), another of four (strains 14, 16, 18, and 19). The remaining seven strains stood alone. Finally, grouping of strains on the basis of results for roughness (Figure 3C) allowed the strains to be separated into 11 clusters, whilst grouping by maximum thickness put them into 9 clusters (Figure 3D).

## 4. Discussion

### 4.1. Prevalence of L. monocytogenes in Poultry

Several studies have shown that poultry meat is one of the foods most often a vehicle for *L. monocytogenes* [14,18]. The prevalence of *L. monocytogenes* detected in the current research (7.7%) lies within the wide range of values observed by other authors, varying from 0% to 58% of samples [1,19,20,21]. However, the percentage of positive samples detected is lower than what was seen in the same geographical area in 1993 (32.0%) [14] and in 2006 (24.5%) [22]. These findings suggest that hygiene measures introduced over the last few years in the European Union have been effective in reducing the prevalence of *L. monocytogenes* in poultry meat.

### 4.2. Serotyping and Ribotyping of Listeria monocytogenes

Several of the serotypes to which strains were ascribed are among those most often associated with processes of human listeriosis, such as 4b, 1/2a, and 1/2b, involved in 95% of cases and of outbreaks of the disease [23]. Other serotypes were also detected that show considerable persistence in food-processing environments, specifically those belonging to lineage II (1/2a, 1/2c, and 3a). The detection in poultry of serotypes involved in events of human listeriosis coincides with the findings of other researchers [1]. This fact is a cause for concern and highlights the need for proper handling of such foods (avoiding cross-contamination and ensuring thorough cooking) so as to reduce the risks of listeriosis for consumers.

Through ribotyping, the twenty strains were grouped into eight clusters (Figure 1). Some authors hold that the ribotyping method has the potential to differentiate serotypes of *L. monocytogenes* [24]. However, in the present work, it proved to be the case that several clusters included strains of more than one serotype. Moreover, ribotyping did not permit the separation of strains of different lineages. Thus, in clusters 1 and 3 strains from both lineage I and lineage II were included.

It has been observed that the discriminatory power of ribotyping using the *Eco*RI enzyme has failed to be adequate in epidemiological studies, especially in respect of isolates belonging to serotype 4b [25,26,27]. It has been suggested that the use of other restriction enzymes might improve the discriminatory power of the technique [28]. In the research being reported here, ribotyping permitted discrimination of strains in the same serotype in various cases, including the four strains of serotype 4b, which it proved possible to assign to three different clusters (3, 7, and 8).

The fact that strains of differing origins were grouped into a relatively small number of clusters underlines the fact that there is a strong genetic relationship among strains of *L. monocytogenes* from different sources in northwest Spain. A similar situation has previously been observed in the case of *Salmonella* [29] and vancomycin-resistant enterococci [30].

### 4.3. Study of Biofilms

This research involved studying biofilms of twenty strains of *L. monocytogenes* incubated under stable conditions at a temperature of 37 °C for 24 h in TSB. The contact surface used was a polystyrene microtiter plate. This plastic is a hydrophobic material frequently used in food-processing installations.

Few data are available relating to intra-specific diversity in *L. monocytogenes* biofilms. Among the different structures seen are layers one cell thick, flat layers multiple cells in thickness without a given structure, networks of interwoven clumps and honeycomb structures [31,32,33,34,35,36]. In the work reported here, it was observed that the biofilms produced took on a multi-layer structure that in some instances came to cover the greater part of the wells in the polystyrene plate, while in others the wells were scarcely colonized. The biovolume observed (running from 621.7 ± 36.0 µm^3^ to 62,984.0 ± 14,888.2 µm^3^) was less than noted in previous studies under the same experimental conditions, using other *L. monocytogenes* strains of food origin (between 103,928.3 ± 6730.2 μm^3^ and 276,030.9 ± 42,291.9 μm^3^) [35,36].

Consideration was given to the influence of the phylogenetic group on the ability of *L. monocytogenes* to form biofilm. *L. monocytogenes* is divided into four evolutionary lineages (I, II, III, and IV), differing in their distribution and prevalence in the environment [37,38]. Strains from lineage I, which includes serotypes 1/2 b, 3b, 3c, and 4b, are the most prevalent in human clinical isolates [39]. However, it is unusual for them to appear in food-processing installations, where strains belonging to lineage II, comprising serotypes 1/2a, 1/2c, and 3a [15] are more persistent. A majority of food isolates of *L. monocytogenes* are from this lineage. Lineages III and IV are uncommon, including serotypes 4a, 4c, and atypical cells of serotype 4b, their presence being linked exclusively to animals and are not typically of clinical relevance for humans [2,37,40].

Some authors have noted that the greater prevalence of strains from lineage II in food-processing contexts might be related to their greater capacity for forming biofilm [41,42,43,44,45]. Nevertheless, other studies have encountered larger biovolumes among strains from lineage I than in those of lineage II [46,47]. In the present research the considerable biomass of the biofilm formed by strain 10 (serotype 3c; lineage I) was noteworthy. Nonetheless, serotypes belonging to lineage II (1/2a, 1/2c, 3a) achieved similar values for biovolume (*p* > 0.05) to serotypes from lineage I (1/2b, 3b, 4b). Hence, this study found no differences between the various lineages in respect of their ability to form biofilm.

Various researchers have also assessed the influence of the serotype upon the capacity of *L. monocytogenes* to form biofilm. The value of studying differences among serotypes is clear. Whilst there are various serotypes of *L. monocytogenes* that can cause disease, only three (1/2a, 1/2b, and 4b) are usually involved in human listeriosis events [6,23]. It should be noted that an important outbreak of listeriosis produced by a chilled roasted pork meat product, that occurred in Spain during August and September 2019, has been linked to *L*. *monocytogenes* serotype 4b [48,49]. Kadam et al. [50] observed that after incubation in TSB at 37 °C (similar conditions to those tested in the present work) serotypes 1/2b and 1/2a formed stronger biofilms on polystyrene than did serotype 4b. The findings of these authors do not coincide with the results obtained in this investigation, where the three serotypes formed biofilms of similar biovolume (*p* > 0.05). The present results do not concur with those of Norwood and Gilmour [31], either. These authors noted that serotype 1/2c formed more biofilm than serotypes 1/2a and 4b, whilst in the current work strains of serotype 1/2c presented very low values for biovolume and percentage of surface covered. Thus, they had only a sparse capacity for forming biofilm.

Folsom et al. [51] indicated that in undiluted TSB medium serotype 4b formed more biofilm than serotype 1/2a. Nevertheless, in diluted TSB serotype 1/2a formed more biofilm than serotype 4b. This may explain why serotype 1/2a is more often found than serotype 4b in food industry environments, where the presence of nutrients is limited [43].

When strains are considered individually, major differences (*p* < 0.05) were observed in the biovolumes of strains of the same serotype (strains 10 and 11, serotype 3c). The same conclusions emerge on scrutinizing Figure 3A, which shows the grouping of strains on the basis of their biovolume. Thus, it will be observed that there are strains sharing the same serotype, yet falling into different clusters. This occurs with serotypes 1/2a, 1/2b, 3b, 3c, and 4b. On these lines, Folsom et al. [51] noted that two isolates of the same strain (Scott A) obtained from different laboratories showed differing levels of formation of biofilm. These findings suggest that the ability to form biofilm under given environmental conditions depends on the strain, rather than on the serotype, and provide a possible explanation for the different results obtained by various researchers when studying the relationship between the capacity to form biofilm and the serotype of isolates.

In respect of percentage of surface covered, it has been observed that on abiotic hydrophobic surfaces, like that used in this work (polystyrene), there is a direct relationship between initial adhesion by the bacterial cells and the volume of biofilm they produce [52]. This could provide support for the correlation between biovolume and the percentage of surface covered. This is because the greater the initial adhesion by bacteria—or, what comes to the same thing, surface area colonized by them—the greater the biovolume produced by the strain involved will be. This claim is borne out by the data obtained. As with biovolume, no significant differences were observed, either between phylogenetic groups or between serotypes, in the percentage of surface covered by biofilms.

Roughness and maximum thickness were the structural parameters with most significant differences among strains, and yet they were not related to phylogenetic or serotype features. Both parameters were directly proportional to the heterogeneity of biofilms. In this research, as mentioned above, inverse correlations were found between roughness and biovolume, and between roughness and percentage of surface covered. Hence, the strains producing the lowest levels of biofilm were those presenting the greatest heterogeneity. Heterogeneity is clearly demonstrated in strains 5 and 12, which had the highest values for roughness and maximum thickness. Their three-dimensional images show large areas of the well completely lacking biofilm, in contrast with areas having biofilm that project a long shadow, thanks to the maximum thickness they have reached (Figure 2).

Use of the values for percentage of surface covered, roughness, and maximum thickness to draw up tree diagrams grouped the strains into 9, 11, and 9 clusters, respectively. This allowed the separation of strains of the same serotype. These results suggest that structural parameters of biofilms could be useful in epidemiological studies. However, further research is needed to support these findings.

## 5. Conclusions

To sum up, the serotypes of *L. monocytogenes* most often implicated in single cases and outbreaks of human listeriosis (1/2a, 1/2b, and 4b) were detected in poultry meat. This suggests that such foods play a major part in transmitting the disease and underlines how important it is for those handling these foodstuffs to apply best practice in hygiene so as to reduce the risk of listeriosis for consumers. In some instances, the ribotyping method used allowed the separation of strains of *L. monocytogenes* with the same serotype. However, it would be beneficial to trial other restriction enzymes that might permit improvements in the discriminatory capacity of the technique. All the strains of *L. monocytogenes* tested were able to form biofilm on polystyrene surfaces. This is a worrying fact for public health, in view of the extensive use of this material in the food industry and the health system. Marked differences were observed between strains in respect of their ability to form biofilm. No significant differences were found between phylogenetic groups (lineages) or serotypes with regard to the ability of *L. monocytogenes* to form biofilm, which suggests that biofilm-forming capacity is a feature associated with strain. A positive correlation was observed between biovolume and percentage of surface covered. These two parameters in their turn correlated negatively with roughness, which indicates that the biofilms with smaller biomasses were the most heterogeneous.

## Figures and Tables

**Table 1:** Structural parameters of biofilms formed by 10 strains of *Listeria monocytogenes* isolated from poultry meat

Strain (Serotype)	Biovolume (µm^3^)	% Surface Coverage	Roughness	Maximum Thickness (µm)
**1 (1/2a)**	12,099.1 ± 12,156.7 a	20.09 ± 15.25 ab	0.434 ± 0.048 a	11.33 ± 2.31 ab
**2 (1/2a)**	2877.1 ± 827.2 a	7.79 ± 2.16 a	0.485 ± 0.029 ab	10.33 ± 0.58 ab
**3 (1/2b)**	28,184.0 ± 21,540.5 a	50.50 ± 31.20 ab	0.548 ± 0.131 ab	19.00 ± 7.00 ab
**4 (1/2b)**	2208.5 ± 2014.4 a	4.05 ± 0.57 a	0.661 ± 0.168 ab	16.33 ± 8.74 ab
**5 (1/2c)**	2600.3 ± 3367.7 a	3.18 ± 1.82 a	0.739 ± 0.153 ab	24.00 ± 14.93 a
**6 (1/2c)**	1225.4 ± 1097.4 a	2.17 ± 0.84 a	0.714 ± 0.031 ab	17.00 ± 2.65 ab
**7 (3a)**	4504.2 ± 2780.8 a	10.38 ± 0.81 a	0.606 ± 0.054 ab	19.33 ± 2.31 ab
**8 (3b)**	18,568.1 ± 4367.1 a	53.85 ± 3.96 ab	0.455 ± 0.033 ab	10.67 ± 2.08 ab
**9 (3b)**	24,234.6 ± 34,507.0 a	37.64 ± 46.81 ab	0.417 ± 0.079 a	11.00 ± 1.73 ab
**10 (3c)**	62,984.0 ± 14,888.2 b	94.43 ± 3.97 b	0.399 ± 0.052 a	14.33 ± 1.53 ab
**11 (3c)**	2054.5 ± 780.5 a	9.97 ± 3.29 a	0.433 ± 0.048 a	10.33 ± 1.53 ab
**12 (4a)**	1725.9 ± 991.6 a	2.52 ± 0.82 a	0.830 ± 0.022 b	24.00 ± 5.00 a
**13 (4a)**	5032.7 ± 2535.7 a	18.39 ± 9.10 a	0.592 ± 0.172 ab	15.67 ± 4.16 ab
**14 (4b)**	18,870.8 ± 8290.3 a	35.92 ± 13.52 ab	0.401 ± 0.013 a	12.00 ± 2.00 ab
**15 (4b)**	1901.6 ± 517.9 a	9.68 ± 2.58 a	0.415 ± 0.026 a	9.00 ± 0.00 b
**16 (4b)**	8059.7 ± 3897.1 a	28.71 ± 11.62 ab	0.452 ± 0.035 ab	10.00 ± 1.00 ab
**17 (4b)**	23,727.1 ± 15,800.7 a	66.49 ± 30.47 ab	0.443 ± 0.039 a	10.67 ± 2.52 ab
**18 (4c)**	19,969.7 ± 1398.8 a	35.42 ± 4.74 ab	0.417 ± 0.005 a	14.00 ± 1.00 ab
**19 (4d)**	13,498.3 ± 1635.5 a	29.30 ± 5.38 ab	0.417 ± 0.006 a	11.67 ± 1.15 ab
**20 (4d)**	621.7 ± 36.0 a	2.27 ± 0.28 a	0.578 ± 0.057 ab	12.00 ± 1.00 ab

Data in the same column sharing a letter show no significant differences one from another (*p* > 0.05). Each value is the result of nine measurements.

**Table 2:** Structural parameters of biofilms formed by strains of *Listeria monocytogenes* of 10 different serotypes.

Serotype	Biovolume (µm^3^)	% Surface Coverage	Roughness	Maximum Thickness (µm)
**1/2a (*n* = 2)**	7488.1 ± 9214.2 a	13.94 ± 11.85 ab	0.460 ± 0.045 ab	10.83 ± 1.60 a
**1/2b (*n* = 2)**	15,196.3 ± 19,739.2 ab	27.27 ± 32.20 ac	0.605 ± 0.148 ac	17.67 ± 7.23 ab
**1/2c (*n* = 2)**	1912.9 ± 2363.4 a	2.67 ± 1.39 a	0.727 ± 0.099 c	20.50 ± 10.33 b
**3a (*n* = 1)**	4504.2 ± 2780.8 a	10.38 ± 0.81 ab	0.606 ± 0.054 bc	19.33 ± 2.31 ab
**3b (*n* = 2)**	21,401.4 ± 22,216.1 ab	45.74 ± 31.01 bc	0.436 ± 0.058 ab	10.83 ± 1.72 a
**3c (*n* = 2)**	32,519.3 ± 34,678.9 b	52.20 ± 46.37 c	0.416 ± 0.049 b	12.33 ± 2.58 ab
**4a (*n* = 2)**	3379.3 ± 2499,1 a	10.46 ± 10.44 a	0.711 ± 0.170 c	19.83 ± 6.15 b
**4b (*n* = 4)**	13,139.8 ± 11,904.2 ab	35.20 ± 26.15 ac	0.428 ± 0.033 b	10.42 ± 1.83 a
**4c (*n* = 1)**	19,969.7 ± 1398.8 ab	35.42 ± 4.74 ac	0.417 ± 0.005 ab	14.00 ± 1.00 ab
**4d (*n* = 2)**	7060.0 ± 7128.3 a	15.78 ± 15.19 ab	0.497 ± 0.095 ab	11.83 ± 0.98 ab

Data in the same column sharing a letter show no significant differences one from another (*p* > 0.05).
